# Statistics of Interest

**Published:** 1891-01

**Authors:** 


### STATISTICS OF INTEREST.

A German statistician says : There are, at present, 3,064 languages spoken by the inhabitants of our globe, whose religious convictions are divided between 1,000 different confessions of faith. The number of males is nearly equal to that of the females. The average duration of life is 33 years. One-fourth of the population of the earth dies before attaining the 17th year. Of 1,000 persons only one reaches the age of 100 years, and not more than six that of 65 years. The entire population of the globe is upward of 1,200,000,000, of whom 32,214,000 die every year; 96,480 every day; 4,020 every hour; 67 every minute, and one and a fraction every second. On the other hand the births amount to 36,792,000 every year, 100,800 every day; 4,200 every hour; 70 every minute; one and a fraction every second. Married people live longer than the unmarried, the temperate and industrious longer than the gluttons and idle, and civilized nations longer than the uncivilized. Tall persons enjoy a greater longevity than small ones. Women have a more favorable chance of life before reaching their fiftieth year than men, but a less favorable one after that period. The proportions of married persons to single ones is as 75 to 1,000. Persons born in spring have a more robust constitution than those born in other seasons. Births and deaths occur more frequently at night than in the day time. Only one-fourth of the male inhabitants of the globe grow up to carry arms or perform military service.

